# Quantum Random Access Memory for Dummies

**DOI:** 10.3390/s23177462

**Published:** 2023-08-28

**Authors:** Koustubh Phalak, Avimita Chatterjee, Swaroop Ghosh

**Affiliations:** School of Electrical Engineering and Computer Science, The Pennsylvania State University, State College, PA 16802, USA; amc8313@psu.edu

**Keywords:** quantum computing, quantum RAM, qudit, bucket-brigade QRAM, flip-flop QRAM, EQGAN, PQC

## Abstract

Quantum Random Access Memory (QRAM) has the potential to revolutionize the area of quantum computing. QRAM uses quantum computing principles to store and modify quantum or classical data efficiently, greatly accelerating a wide range of computer processes. Despite its importance, there is a lack of comprehensive surveys that cover the entire spectrum of QRAM architectures. We fill this gap by providing a comprehensive review of QRAM, emphasizing its significance and viability in existing noisy quantum computers. By drawing comparisons with conventional RAM for ease of understanding, this survey clarifies the fundamental ideas and actions of QRAM. QRAM provides an exponential time advantage compared to its classical counterpart by reading and writing all data at once, which is achieved owing to storage of data in a superposition of states. Overall, we compare six different QRAM technologies in terms of their structure and workings, circuit width and depth, unique qualities, practical implementation, and drawbacks. In general, with the exception of trainable machine learning-based QRAMs, we observe that QRAM has exponential depth/width requirements in terms of the number of qubits/qudits and that most QRAM implementations are practical for superconducting and trapped-ion qubit systems.

## 1. Introduction

Quantum Computing (QC) has progressed rapidly in the past decade. With the advancement in qubit technologies such as superconducting qubits [1], trapped ion qubits [2], photonic qubits [3], quantum dots [4], and diamond nitrogen-vacancy centers [5], implementation of quantum algorithms on quantum computers has become practically possible. This has enabled the application of quantum computing in fields such as machine learning [6], finance [7], chemistry [8], cybersecurity [9], and advanced manufacturing [10]. A potential game changer in quantum computing is the augmentation of Quantum Random Access Memory (QRAM), which has shown potential to provide exponential speedup for Fourier transform [11], discrete logarithm [12], and pattern recognition [13,14,15] algorithms. QRAM is a key requirement for important quantum algorithms such as quantum searching of classical databases [16,17], collision-finding for hash and claw-free functions [18], and distinctness of elements in a list [19,20]. Along with this, QRAM can serve as an important memory element to load classical data into the quantum Hilbert space as compared to simpler methods such as amplitude, angle, and basis embeddings [21].

The existing literature on QRAM fails to summarize key aspects of QRAM and explain them in layman’s terms, which is the objective of this paper. In [22], the authors discussed various QRAM approaches such as bucket-brigade QRAM, large width–small depth QRAM, and small width–large depth QRAM; however, they approached the topic from a fault-tolerance standpoint rather than a fundamental explanatory perspective. An overview of the practicality of QRAM in modern Noisy Intermediate-Scale Quantum (NISQ) systems is provided in [23]; however, it can be esoteric at times and difficult to fully comprehend. In this paper, we provide a simple-to-grasp review of QRAM for readers interested in diving deeper into the field of quantum memory. While complex mathematical knowledge of quantum physics is not required, we do assume that the readers know the fundamentals of quantum computing [24], such as ket notation, quantum gates, and quantum circuit notation.

We perform a thorough review by first presenting important information on QRAM, then discuss each QRAM technology in turn. For each different approach to QRAM, we describe its structure, the circuit width and the circuit depth, and with its unique qualities. Finally, we talk about the practical implementation of QRAM and weigh the pros and cons through a tabular comparison. This review considers six QRAM technologies that have been published in the literature within period ranging from 2008 to 2022. These works include Bucket-Brigade QRAM [25], Fan-Out QRAM [26], Flip-Flop QRAM [27], Qudits-based memory [28], Approximate PQC-based QRAM [29], and EQGAN-QRAM [30].

The remainder of this paper is organized as follows: in Section 2, we provide preliminaries on quantum computing and the workings of classical RAM; in Section 3, we delve into the fundamentals of QRAM, answering key questions about its structure, utility, and requirements; Section 4 explores the practical implementation of QRAM; and Section 5 offers an overview of the challenges involved in implementing QRAM and its future potential. Finally, we conclude the paper in Section 6.

## 2. Preliminaries

### 2.1. An Overview of Quantum Computing

#### 2.1.1. Qubits

Qubits, the elementary units of quantum computing, are distinct from classical bits in that they can exist in a superposition of states and represent both 0 and 1 simultaneously. This unique property allows quantum computers to perform multiple computations in parallel, providing the potential for exponential speedup compared to classical computers. In a Hilbert space, a qubit is represented by a two-dimensional vector denoted as |ψ〉=α|0〉+β|1〉, where α and β are the coefficients of the basis states of the qubit. These coefficients are constrained by the normalization condition ${\left|\alpha \right|}^{2}+{\left|\beta \right|}^{2}=1$, and the probabilities of measuring the state of the qubit in the basis state of |0〉 or |1〉 are provided by ${\left|\alpha \right|}^{2}$ or ${\left|\beta \right|}^{2}$, respectively [24].

#### 2.1.2. Quantum Gates

Quantum gates are the fundamental operations that act on qubits in a quantum circuit, akin to how classical logic gates operate on classical bits. These gates include the Pauli-X, Pauli-Y, Pauli-Z, Hadamard, CNOT, and Toffoli gates. They are often depicted as unitary matrices that act on qubit states to maintain the quantum properties of the system [31]. Quantum gates can be created and realized physically utilizing a variety of techniques, including lasers, magnetic fields, and microwave pulses [24].

#### 2.1.3. Quantum Circuit

Quantum circuits are collections of quantum gates that work together to carry out particular quantum computations. Initialization of the qubits is the first step in the creation of a quantum circuit. Gate operations, which involve multi-qubit gates such as the CNOT and the Toffoli gates as well as single-qubit gates such as the Hadamard and Pauli gates, are used to change the qubits to the required state. Prior to execution, the high-level gates in the circuit, including the Toffoli gate, are disassembled into a native gate set of the quantum hardware (called transpilation). The output of the quantum circuit is then obtained by measuring the qubits using a measurement gate, which converts the quantum state into a classical state [32].

#### 2.1.4. Quantum Entanglement

Two or more qubits can become correlated in a way that prevents one qubit from being described independently of the other qubits through the phenomenon known as quantum entanglement. This characteristic is critical for the development of effective quantum algorithms and protocols, including quantum teleportation and superdense coding [33]. The nonlocal correlations available through entanglement are crucially importance in quantum computing, as they permit the execution of tasks that are classically impossible.

#### 2.1.5. Quantum Superposition

**Figure 1:**
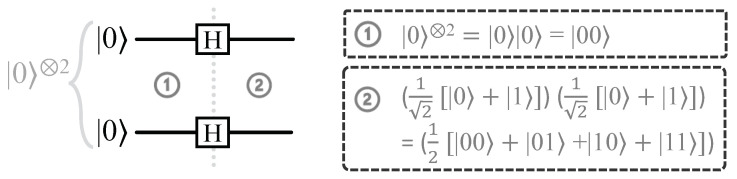
The presented circuit illustrates a fundamental instance of quantum superposition. It commences with an initial 2-qubit state **①** and culminates in a superposition state **②**, demonstrating the essential properties of quantum systems. **①**: |0〉|0〉=|00〉; **②**: $\frac{1}{2}\left[|00\rangle +|01\rangle +|10\rangle +|11\rangle \right]$.

Superposition is a phenomenon that allows both of the computational basis states |0〉 and |1〉 to exist in the quantum Hilbert space at the same time. A qubit state can be placed into superposition using the Hadamard (H) gate. If the initial qubit state is |0〉 (|1〉), then the superposition state after the H gate becomes $\frac{1}{\sqrt{2}}\left(|0\rangle +|1\rangle \right)$ ($\frac{1}{\sqrt{2}}\left(|0\rangle -|1\rangle \right)$). We present an example in Figure 1 showing how to generate a superposition of all the basis states for a two-qubit system.

#### 2.1.6. Quantum Algorithms and Applications

The potential of quantum computing has been illustrated by a number of quantum algorithms. Examples include Grover’s method for exploring unsorted databases [16], Shor’s algorithm for factoring large integers [12], and the quantum simulation algorithms [34]. Among other applications, these algorithms have substantial effects on cryptography, optimization, and quantum system simulation. Combinatorial optimization issues can be resolved using variational quantum–classical algorithms such as the Quantum Approximate Optimization Algorithm (QAOA) [35], Variational Quantum Eigensolver (VQE) [36], and Quantum Machine Learning (QML) models such as Quantum Support Vector Machine (QSVM) [37] and Quantum Principal Component Analysis (QPCA) [6].

### 2.2. Classical RAM

Desktop computers can often slow down when running data-intensive applications. To address this issue, one solution is to install additional Random Access Memory (RAM), which can provide a temporary storage medium for the central processing unit (CPU) to retrieve data quickly in any order while executing a program. RAM is volatile ‘read/write’ memory that stores data temporarily while the computer is operational. When the computer is switched off, these stored data are lost due to their volatile nature. RAM is more efficient than hard drive storage for temporary storage due to its faster access time. The fundamental capability of any computing device is the ability to store and manipulate information in a series of memory cells organized in an array [38]. RAM is the most well-known architecture for such a memory array, as it allows each cell to be addressed [39].

**Figure 2:**
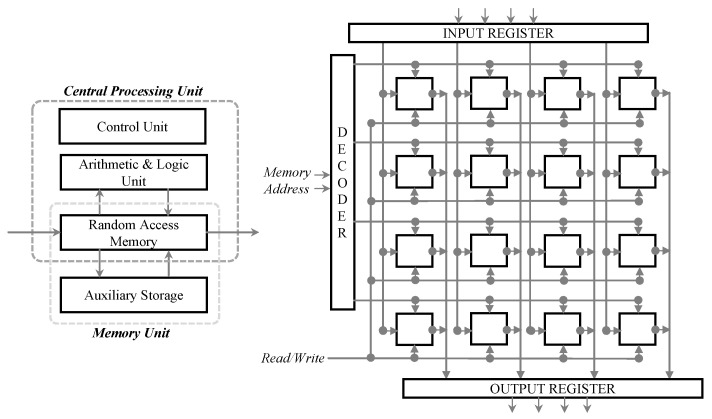
***Left:*** Depiction of the placement of RAM within the memory hierarchy, highlighting its proximity to the CPU. The speed of RAM can be attributed to this closeness, as it serves as an intermediary between the CPU and auxiliary memory systems. ***Right:*** A detailed representation of the various functional components within the RAM, illustrating their organization and interconnections. *Memory Array:* made up of a grid of rows and columns that stand in for memory cells used to store data; one piece of information is stored in each cell. *Input Register:* during a write operation, it temporarily stores the data that are stored in the memory array. *Output Register:* during a read operation, it temporarily stores the data that were read from the memory array. *Decoder:* takes the memory address from the address bus and converts it into row and column coordinates, allowing it access to the associated memory cell. To demonstrate the flow of row and column signals, the arrows from the decoder should point in the direction of the memory array. *Control Bus:* transmits read and write enable signals to the memory array to control data access activities.

A memory array, an input register, and an output register constitute RAM. The memory cells are organized into rows and columns, with each cell holding one bit of data. Data are accessed and manipulated using address lines, data lines, and control lines (read and write enable signals). When the CPU needs to access data from the memory, it sends the memory address through the address lines. Depending on the read or write signal, the data are either retrieved from the memory cell (read operation) or stored in the memory cell (write operation) [40]. The contents of a memory cell are recovered and transferred to the output register when the address of that cell is loaded into the address register, a procedure known as ‘decoding’. Traditional RAM requires effective data storage, retrieval, and manipulation in order to function. The two main types of RAM, namely, Static Random Access Memory (SRAM) and Dynamic Random Access Memory (DRAM), have unique characteristics that determine their use in different applications [41]. Figure 2 illustrates the position of RAM within the memory hierarchy and presents a functional block diagram showcasing its key components and their interactions.

## 3. Fundamentals of QRAM

QRAM is a memory element analogous to RAM that is able to store data in a quantum format. Similar to RAM, QRAM has three components: the input (or address) register, the output (or data) register, and the memory arrays. The difference here though is that the input and output registers are composed of qubits instead of bits, while the memory arrays can be either classical or quantum depending on the use of QRAM [25]. For example, for the two fan-out QRAM implementations in [26], the optical implementation has 1-bit classical memory cells that change the polarization of the output register photons based on the bit value while the phase gate implementation uses two superconducting qubits in a single memory cell (one for storing information and one for extracting information). Table 1 shows the differences between RAM and QRAM. Another key difference in QRAM is the way in which memory access is performed. Rather than accessing a single memory location at a time, QRAM uses superposition to simultaneously access multiple memory locations. This is made possible by leveraging the power of quantum Hilbert space, where all memory addresses are first loaded into superposition. The overall state is then passed through the QRAM to obtain another superposition state, this time with the addresses and data combined. Say that we have *n* qubits, and consequently have $N=2^{n}$ address lines. All the addresses are represented as basis states, from |0〉 to |N−1〉 [25], and stored in the address register *r*. Each address |i〉 has an amplitude $\alpha_{i}$; thus, the effective superposition of the addresses is $\sum_{i=0}^{N-1}\alpha_{i}{|i\rangle }_{r}$. This superposition state is then sent to QRAM and the output is another superposition state, which contains both the address state and the data state chosen from the data register *o*. If $X_{i}$ represents the data in address *i*, then the output state of the QRAM is $\sum_{i=0}^{N-1}\alpha_{i}{|i\rangle }_{r}{|X_{i}\rangle }_{o}$. Effectively, the storage of data in QRAM can be summarized through the following equation:

$$\begin{array}{cc} \; & \sum_{i=0}^{N-1}\alpha_{i}{|i\rangle }_{r}\overset{\mathrm{QRAM}}{\to }\sum_{i=0}^{N-1}\alpha_{i}{|i\rangle }_{r}{|X_{i}\rangle }_{o} \end{array}$$

However, retrieving the data can be challenging due to the no-cloning theorem [42]. This is generally dealt with by performing entanglement operations between memory cell qubits and output register qubits using gates such as the SWAP gate or CNOT gate.

From the aforementioned statements, readers may become curious and ponder several crucial aspects of QRAM, such as (i) the motivation behind the need for QRAM: *Why do we need QRAM?*; (ii) the configuration of QRAM: *What is the structure of QRAM?*; and (iii) the extent of QRAM’s utility and usage: *Where is a QRAM used?* We provide answers to all of these questions in the following subsections.

### 3.1. Why Do We Need QRAM?

In quantum computing, the fundamental building blocks of computation are quantum states, which can represent information as a superposition of basis states. These quantum states are fragile, and are sensitive to external disturbances such as environmental noise and decoherence [43] that can cause them to rapidly lose coherence and become unusable for computation. Present-day quantum computers are plagued by noise. These noisy quantum computers are formally known as NISQ computers, which refers to the susceptibility of quantum computing technology to qubit errors caused by varied sources of noise, including thermal fluctuations, electromagnetic interference, and device imperfections. Errors such as decoherence, cross-talk, gate errors, etc., can degrade the overall fidelity of computation. In such an environment, loading data in the quantum Hilbert space can be challenging due to its large gate and circuit depth requirements. The overall loading process can end up being noisy, and may store inaccurate data as a result. QRAM can provide data reliably if it is implemented using shallow gate count and low depth circuits. By storing data in superposition, QRAM can enable parallel data access, which is important for efficient use of quantum algorithms. This parallel data access reduces the overall access time, increasing resilience against noise. Therefore, it is crucial to be able to efficiently store and retrieve quantum states themselves in order to execute quantum algorithms.

Classical memory devices are not suitable for storing quantum states, as they requires collapsing of the wave function through a measurement operation [44]. The collapse of the wavefunction destroys the superposition of states, and causes the quantum state to take on a singular classical value (either 0 or 1), which can be stored in classical RAM but is no longer be valuable for quantum computation. QRAM is a potential solution to this problem, as it allows quantum states to be stored and retrieved efficiently without collapsing the superposition of states. This is accomplished by using quantum mechanics to encode information in a way that is resistant to decoherence and other sources of noise [45]. This allows quantum states to be stored and retrieved with minimal error, making QRAM an essential component of quantum computing technology.

In addition, QRAM can potentially be useful for loading classical data into quantum Hilbert space. Hybrid quantum–classical optimization algorithms in the field of QML often require the conversion of classical data in Euclidean space (e.g., image datasets such as MNIST, Iris, CIFAR-10/100, etc.) into to quantum data in Hilbert space. This is achieved using encoding methods such as angle embedding, amplitude embedding, and basis embedding [21]. Amplitude embedding embeds $2^{n}$ classical features on *n* qubits, while angle and basis embeddings embed *n* classical features on *n* qubits. A problem with these methods, however, is that they are rather simplistic in nature and do not take the complexity of the dataset into account. QRAM-based data loading can potentially address the above issue.

### 3.2. What Is the Structure of a QRAM?

The different QRAM architectures that have been proposed to date are described in this subsection.

#### 3.2.1. Bucket-Brigade QRAM

The very first proposal of a QRAM [25] implemented a bifurcation graph-based structure as opposed to the traditional d-dimensional lattice of memory arrays (shown in Figure 2). This approach is called bucket-brigade QRAM; the bifurcation graph for this QRAM is a binary tree with the leaf nodes as the memory cells and the rest of the nodes as switches used to route the address state to the correct cell. Overall, there are three main components of this QRAM: the *input register*, the *QRAM itself*, and the *output register*. Note that the terms input/index/address register and output/data register/quantum bus are used interchangeably in the literature. For ease of understanding, in this paper we use the terms input register and output register. There are two primary cases to explain how a bucket-brigade QRAM works; in the first there is only a single address in the input register, while in the second there is a superposition of addresses in the input register. These two cases are explained below.

**Figure 3:**
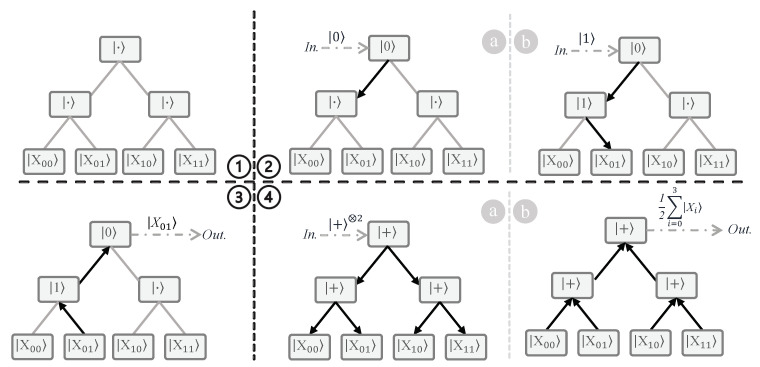
Working of a bucket-brigade QRAM with two address lines and four memory cells. **①** The initial state of the QRAM; all quantum switches are initialized to the |·〉 state, which is a waiting state in which the quantum switch waits for incoming qubit states of the memory address to be accessed. **②** The input register activates switches that allow the output register to access data with address |01〉 ($|X_{01}\rangle$). The address qubits are sent in sequential top-down fashion, from the Most Significant Bit (MSB) to the Least Significant Bit (LSB). In the example shown here, the MSB qubit |0〉 is sent first, and changes the state of the root quantum switch **ⓐ**, followed by the LSB qubit |1〉, which routes the switch in the direction of the memory cell $|X_{01}\rangle$**ⓑ**. **③** The output register reads the data $|X_{01}\rangle$ via the activated quantum switches. **④** The superposition of all addresses turns on all quantum switches **ⓐ** to read the superposition of all the data **ⓑ**. Note that $|+\rangle =\frac{1}{\sqrt{2}}\left(|0\rangle \right)+|1\rangle )$. *In.*: Input register, *Out.*: Output register.

**Single Address Case.** First, consider a QRAM that supports two addresses (two qubits) and uses four memory cells for storage. The initial bifurcation graph for this QRAM is shown in Figure 3, with the quantum switches initialized at the wait state and the four memory cells present at the leaf nodes. Each quantum switch is a three-level system with states |·〉, |0〉, and |1〉, unlike a qubit, which is a two-level system (|0〉 and |1〉). This three-level system is often referred to as a qutrit, and is inspired by the classical three-level system trit, which is generally a tri-state logic multiplexer [46]. The significance of the wait state |·〉 in each quantum switch is that whenever a qubit state (either |0〉 or |1〉) is received by the switch, it changes from |·〉 to the received state. This helps to ensure that the next time the same switch receives another qubit state it will route the qubit state to one of its children’s node switches. The wait state ensures that unaccessed memory cells are not disturbed. The direction of routing depends on the state of the qubit. Typically, |0〉 (|1〉) routes the next state to the left (right) child.

**Figure 4:**
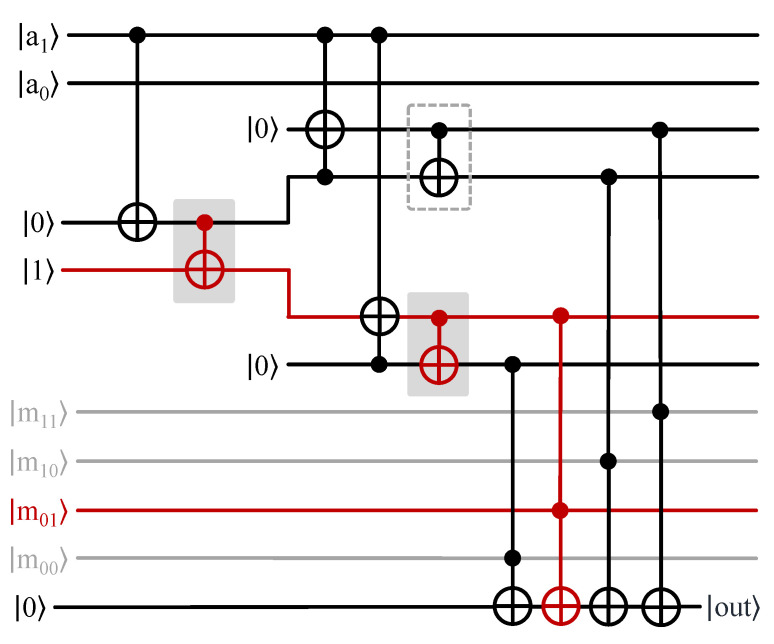
Circuit-based implementation of a bucket-brigade QRAM. Th data in memory cell $m_{01}$ with address |01〉 are being accessed via a series of CNOT and Toffoli gates performing intermediate computation on ancilla qubits. The CNOT gates highlighted in red are the ones being activated, and the red path represents the active route of the QRAM.

Next, we show the example of an incoming address |01〉 accessing the initialized QRAM. The address state comes from the input register in sequential fashion from the Most Significant Bit (MSB) to the Least Significant Bit (LSB). Because the address is |01〉, the MSB is |0〉 and the LSB is |1〉. Therefore, the state |0〉 is first sent to the root node switch of the QRAM. Because the root node switch is in |·〉, it changes its state to |0〉 (Figure 3.2(a)). Next, the LSB state |1〉 arrives at the root node switch, which routes it to the left child. The left child is then activated to state |1〉 (Figure 3.2(b)). In this way, all the address qubits in the input register are used to create a route to the memory cell $|X_{01}\rangle$. Along with the graph-based implementation, we show the circuit-based implementation of the bucket-brigade QRAM with two address lines and four memory cells (inspired from [47]) in Figure 4.

After the creation of the route, the data in the output register can either be stored or read in the routed memory cell. Figure 3.3 shows an example of reading the contents of the address |01〉 ($|X_{01}\rangle$) from the memory cell to the output register along the route. Note that in order to store new data the direction of routing need to be opposite, i.e., from the output register to the memory cell.

**Superposition of Addresses.** In this case, all the qubits are present in a superposition state, similar to the case shown in Figure 1. When a qubit in superposition encounters a quantum switch, the switch changes from |·〉 to the superposition state $|+\rangle =\frac{1}{\sqrt{2}}\left(|0\rangle +|1\rangle \right)$. Because the superposition state includes both the |0〉 state and the |1〉 state, the quantum switch activates both the left and right routes. In this way, the routes to all memory cells are activated when all the superposition address qubits arrive at all the quantum switches (Figure 3.4(a)). When the read operation on the output register is performed, the contents of all the memory cells traverse the activated routes and load a superposition of all the data $\frac{1}{2}\sum_{i=0}^{3}|X_{i}\rangle$ on the output register. Compared to classical RAM, the advantage provided by bucket-brigade QRAM is that whereas the classical RAM requires O($2^{n}$) transistor activations for *n* address lines to access data in a single address, QRAM requires only O(*n*) quantum switch activations. Furthermore, QRAM can read the data from all the addresses at a comparable classical cost of O($2^{n}$) quantum switch activations.

#### 3.2.2. Fan-Out QRAM

A follow-up paper [26] on the original bucket-brigade QRAM work [25] presented architectural implementations of bucket-brigade QRAM along with another QRAM termed ‘fan-out’ QRAM. Fan-out QRAM is taken directly from its classical equivalent, fan-out RAM, where each $k^{th}$ address bit controls $2^{k}$ switches. Usually, for an *n*-bit binary address, the MSB is considered the 0^th^ address bit and the LSB is considered the $n-1^{th}$ address bit. The quantum version of the fan-out RAM has a $k^{th}$ address qubit controlling $2^{k}$ quantum switches. A difference between the quantum switches of bucket-brigade QRAM and the fan-out QRAM is that while bucket-brigade QRAM requires qutrits, fan-out QRAM requires only a two-level system; thus, qubits are used as the quantum switches. Initially, all of the quantum switches are initialized to |0〉 state. Next, the address qubits in the input register are used to change the state of the quantum switches. All of the quantum switches connected to an address qubit in state |0〉 remain at state |0〉, while those connected to an address qubit in state |1〉 change their states to |1〉.

To explain the functionality of fan-out QRAM, we can again consider QRAM with two address lines and four memory cells. The bifurcation graph for QRAM with the quantum switches initialized to the |0〉 state is shown in Figure 5.1. As in the previous case, the input register can contain either a single address or a superposition of addresses.

**Single Address Case.** First, let us take the simple case of single address access, where the memory cell in address |01〉 ($X_{01}$) is being accessed. The MSB address state |0〉 has index 0 and controls the quantum switch $2^{0}=1$, which is the root node switch. The LSB address state |1〉 has index 1; thus, it controls $2^{1}=2$ quantum switches, which are the two child switches of the root node switch. The root node switch stays at state |0〉, while the child switches change their states to state |1〉 (Figure 5.2). As a consequence, all the quantum switches are activated, while only a single complete path from the root node to the memory cell is active (in this case, $X_{01}$). The contents from the memory cell are then either updated from or loaded into the output register via the active path (Figure 5.3).

**Superposition of Addresses.** Extrapolating the above process to a superposition of addresses, the quantum switches are switched to a superposition. As a result of the superposition, each quantum switch activates the routes to both of its child switches, as shown in Figure 5.4. Finally, for a read operation, the contents of all the memory cells traverse all the active routes and the output register reads a superposition of the data, similar to the case shown in Figure 3.4(b). Compared to bucket-brigade QRAM, fan-out QRAM activates O($2^{n}$) switches to access both single addresses and the superposition of all addresses.

#### 3.2.3. Flip-Flop QRAM

A more recent quantum circuit-based QRAM implementation called flip-flop QRAM (FF-QRAM) was proposed in [27]. FF-QRAM stores binary data in superposition one by one, such that the overall circuit has exponential circuit depth and linear width in terms of the number of address lines (or address qubits). Assuming that there are *n* address lines and that the size of the binary data in each address is *m* bits, the QRAM circuit has circuit depth O($2^{n}$) and circuit width O(n+m). Storing a single data point occurs in three stages: the *flip* stage, the *register* stage, and the *flop* stage. The flip stage is a ‘compute’ stage that is used to match all the data and address qubit states to |1〉, which is stored; the register stage consists of a multi-controlled rotation gate that stores the data in a register qubit; and the flop stage is an ‘uncompute’ stage which performs the inverse of the compute stage operation on the address and data qubits.

To explain the working of FF-QRAM, consider a two-address-line QRAM with four address–data pairs, with each datapoint having a size of 2 bits (n=2; m=2). For each of the four addresses, we have the data shown in Table 2, and for each datapoint we generate its respective rotation angle in two steps: (i) first, we normalize the data; if we have data {2,3,1,1}, the normalization factor is $\sqrt{2^{2}+3^{2}+1^{2}+1^{2}}=\sqrt{15}$ and we divide the dataset by the normalization factor, making the new normalized dataset $\frac{1}{\sqrt{15}}\left\{2,3,1,1\right\}=\left\{0.51,0.77,0.25,0.25\right\}$; (ii) second, we compute the rotation angle for each datapoint using the normalized values. The rotation angle $\theta_{k}$ for a datapoint $x_{k}$ with normalized value $x_{k,n}$ is given as $\theta_{k}=2$arcsin($x_{k,n}$) [48]. For the example shown in Table 2, the rotation angles are 2arcsin({0.51,0.77,0.25,0.25}) ={1.06,1.74,0.5,0.5}.

**Figure 6:**
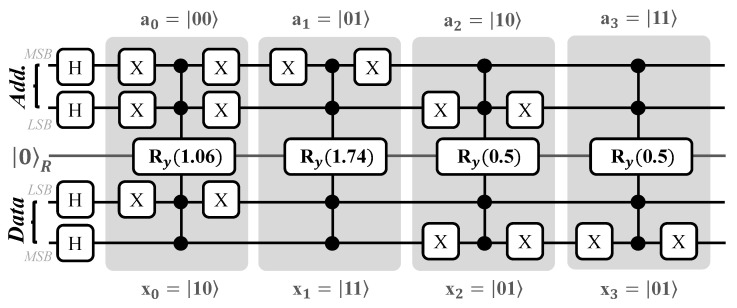
Working of FF-QRAM circuit. The QRAM stores data 10, 11, 01, and 01 in addresses 00, 01, 10, and 11 respectively.

Now that the rotation values have been computed, we can build the FF-QRAM circuit for these address–data pairs. We show the FF-QRAM for our example dataset in Figure 6. The quantum circuit has 2 qubits for address lines, 2 qubits for data lines, and 1 qubits for register lines. First, all of the address and data qubits are initialized to the state |0〉 and brought into superposition using the Hadamard gate. After this, the process of storing the data begins. As mentioned earlier, the datapoints are stored one by one in three stages. In the flip stage, which is the compute stage, the qubit states of the relevant address and data are flipped to |1〉 such that the multi-controlled $R_{y}$ rotation gate is triggered to store the rotation of the desired data. This is achieved using classically-controlled NOT gates [27]. Essentially, when the classical bit value is 0, the NOT gate is activated; otherwise, the NOT gate is not activated. We present a simpler version of this gate here for ease of understanding. When the classical bit value is 0, we place an X gate, while when the classical value is 1 we do not place the X gate. Consider the first address–data pair |00〉−|10〉 in Figure 6. For address qubits, because both the target address lines are in state |0〉, we place an X gate on both of the qubits. Similarly, for the data lines we place an X gate only on the LSB qubit. Next is the register stage, where we add the multi-controlled $R_{y}$ gate with a computed rotation angle of 1.06 radians. Finally, we then add the inverse of the flip stage in the flop stage, in which X gates are placed only on those qubits where X gates were placed during the flip stage. The same process is repeated for all the remaining address–data pairs, as shown in Figure 6. The end result of this repetitive process is that the FF-QRAM has a superposition of addresses and their corresponding data and angles stored in the address, data, and register qubits, respectively.

#### 3.2.4. Entangling Quantum Generative Adversarial Network (EQGAN) QRAM

EQGAN [30] is a pure quantum entanglement-based Generative Adversarial Network (GAN) which is PQC-based and has a quantum generator and a quantum discriminator that are both trained together with a minimax game. For a discriminator model *D* with parameters $\theta_{d}$, generator model *G* with parameters $\theta_{g}$, real data σ, and generated data $\rho (\theta_{g})$, the minimax problem is provided as follows:

$$\begin{array}{cc} \; & \min_{\theta_{g}}\max_{\theta_{d}}C\left(\theta_{g},\theta_{d}\right)=\min_{\theta_{g}}\max_{\theta_{d}}\left\{1-D_{\sigma }\left[\theta_{d},\rho \left(\theta_{g}\right)\right]\right\}. \end{array}$$

**Figure 7:**
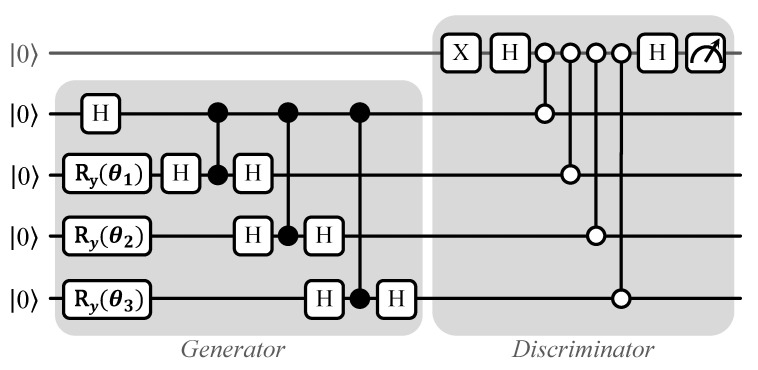
EQGAN variational QRAM circuit for storing superposition of data from class 0; the generator ansatz is only slightly different for storing the superposition of data in class 1.

This EQGAN model is used for variational QRAM as an application in which datapoints from two Gaussian distributions are stored. The QRAM [30] uses two generators with exponential peak ansatz, one for class 0 and one for class 1 (where each class signifies data from one Gaussian distribution), and a swap-test-based discriminator. We show the circuit of this variational EQGAN QRAM, which stores data from class 0, in Figure 7. For class 1, the PQC is nearly the same, with a slight difference in generator ansatz. Using this generator–discriminator setup, the QRAM is able to place data into superposition approximately using constant O(1) gates. Another advantage of this approach to QRAM is observed in classification tasks. Without QRAM, training the data on a Quantum Neural Network (QNN) yields an average classification accuracy of 45%, while when augmented with QRAM the average classification accuracy increases to around 65%.

#### 3.2.5. Qudits-Based Memory

Qudits are higher-state quantum units that contain more than two computational basis states. While a qubit in superposition can be represented as |ψ〉=α|0〉+β|1〉, the superposition of a qudit with *d* computational basis states is represented as

$$\begin{array}{cc} \; & |\psi \rangle =\alpha_{0}|0\rangle +\alpha_{1}|1\rangle +....\alpha_{d-1}|d-1\rangle =\sum_{i=0}^{d-1}\alpha_{i}|i\rangle \end{array}$$

Recent works such as [28,49] have proposed qudit-based quantum memory, in which qubits are temporarily compressed onto qudits in their higher states using reversible compression circuits. When unused, the qudits can be used elsewhere as ancillary bits. During computing, the qudits can be reverted back to qubits by performing the inverse of the compression circuit operation.

The authors of [28] defined two higher state gates analogous to the X-gate. Assuming that the input qubit state is |i〉, (i) the $X_{+t}$ gate performs the operation $|i\rangle \overset{X_{+t}}{\to }|(i+t)\;\mathrm{mod}\;d\rangle$ (ii) and the $X_{ij}$ gate performs $|i\rangle \overset{X_{ij}}{\to }|j\rangle$ and $|j\rangle \overset{X_{ij}}{\to }|i\rangle$. In this approach, there are two qudit-controlled versions of these gates, where the control qudit has a reference control state and the target qudit has the gate. An x-y-z qudit–qubit compression scheme is introduced that is dependent on the radix of the input and output qudits. Here, *x* is the radix of the input qudits, *y* is the radix of the output qudits, and *z* represents the number of ancilla generated. The compression scheme should follow $x^{a}\leq y^{b}$ such that 0<b<a and a−b=z, where *a* and *b* are integers, $x^{a}$ denotes the number of computational basis states of the input, and $y^{b}$ denotes the number of computational basis states of the output. A natural restriction is that the computational basis states of the output should all be higher than that of the input. Another restriction is that the number of input qudits *a* should be greater than the number of output qudits *b*, enabling compression; as a result of this compression, a total of a−b=z ancilla qubits are generated.

**Figure 8:**
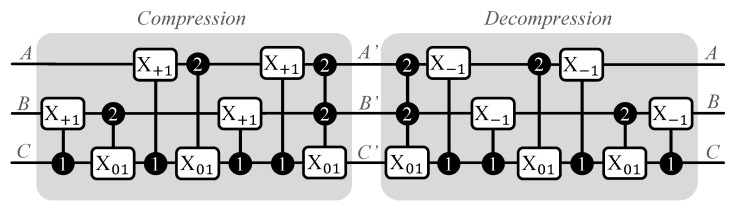
Compression and decompression circuits for a 2-3-1 compression scheme [28]. The compression circuit compresses the contents of three qubits (A,B,C) into two qutrits (A’,B’) and generates a free ancilla in state |0〉 (C’). The decompression circuit then reverts the qutrits and ancilla back to the original three qubits.

A simple example is the conversion of qubits (d=2) to qutrits (d=3). Three qubits can store $2^{3}=8$ computational basis states and two qutrits can store $3^{2}=9$ computational basis states; thus, three qubits can be compressed into two qutrits and the leftover qubit can be generated as an ancilla that can be used in other circuits. In this example, we have x=2, y=3, and z=1; thus, we have a 2-3-1 compression scheme. We show the compression and decompression circuits of this scheme in Figure 8. The compression circuit consists of controlled $X_{+1}$ and $X_{01}$ conditioned on either |1〉 or |2〉 states. The decompression circuit has the gates of the compression circuit in reverse order, with an added difference of having controlled $X_{-1}$ gates instead of controlled $X_{+1}$ gates. The truth table of the 2-3-1 compression scheme is shown in Table 3. By substituting the values of the qubits A, B, and C in the circuit and performing higher-level qudit operations, it can be verified that the compression yields corresponding A’, B’ (qutrits), and C’ (ancilla in |0〉 state) entries from the truth table and that decompression yields back the original values of A, B, and C.

#### 3.2.6. Approximate PQC-Based QRAM

**Figure 9:**
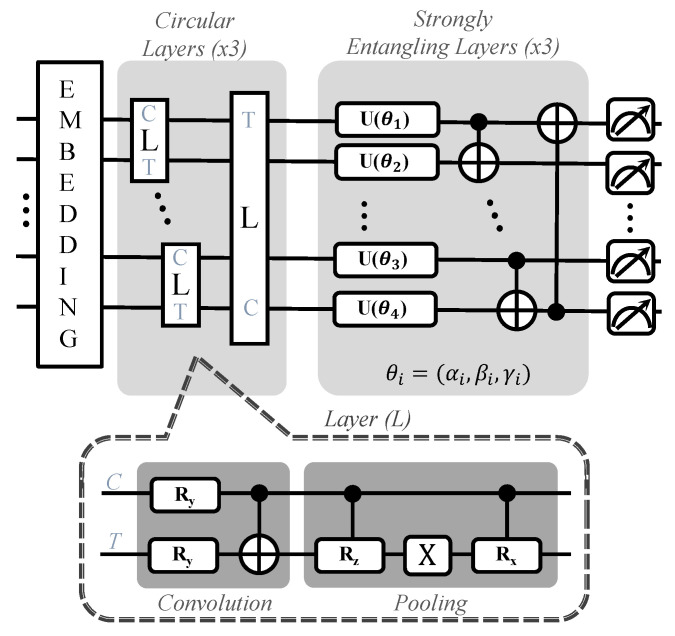
PQC structure of approximate PQC-based QRAM. In the context shown here, ‘C’ symbolizes the control qubit and ‘T’ stands for the target qubit.

In [29], a trainable PQC-based QRAM similar to EQGAN QRAM was proposed that is able to store data in the quantum Hilbert space by training the PQC. Compared to EQGAN, approximate PQC-based QRAM does not store data in a superposition, instead using a one-by-one in sequential order; thus, it is able to store more complex datasets such as image datasets such as the UCI digits dataset. approximate PQC-based QRAM can be used for the storage of purely binary data as well. The detailed PQC of the approximate QRAM is shown in Figure 9. It consists of an embedding scheme, such as angle, amplitude, or basis embedding, used to load classical data, followed by three sets of circular layers and strongly entangling layers. It has been noted that loading images from QRAM and sending them to a QNN yields faster convergence of classification (by the 6th epoch) as compared to loading images without QRAM (around the 15th epoch), and for pure storage the QRAM is able to store 4-bit binary data without any errors.

### 3.3. Where Is QRAM Used?

QRAM that is able to store and load data in superposition is very helpful for certain classes of quantum algorithms.

- *Database search:* Grover’s algorithm [16], along with its more generalized version, Quantum Amplitude Amplification and Estimation (QAE) [17], have been proposed to perform database search for an element out of *n* elements with complexity O($\sqrt{n}$). They take data in superposition as the input and perform the amplification operation O($\sqrt{n}$) times prior to performing estimation with a reduced number of measurements.
- *Element distinctness:* for a set of *n* elements, the element distinctness problem asks whether all *n* elements in the set are distinct. Classically, this takes O(*n* log(*n*)) time, while quantum algorithms such as [19] can solve it in O($n^{\frac{2}{3}}$) time.
- *Collision detection:* collision detection is an important problem in cryptography. For a given collision function *H*, the collision detection problem asks for two distinct inputs, *x* and *y*, such that H(x)=H(y). Quantum versions of the collision detection problem such as [18] report O($n^{\frac{1}{3}}$) runtime, where *n* denotes the cardinality of the domain of the collision function.
- *NAND tree evaluation:* in this problem, a Boolean expression is solved using a tree of NAND gates. For an input of size *n*, quantum algorithms such as [50] propose a runtime of O($\sqrt{n}$).
- *Quantum forking:* in classical operating systems, forking is the process of creating a child process from a parent process which is a copy of it while retaining the parent process. Quantum forking is a similar idea, in which the QRAM output superposition state is forked onto ancilla qubits and then both the original state and forked state are multiplied by the same or different unknown unitaries. The new states then undergo a swap-test procedure to verify whether the applied unitaries are the same or different [27,51].
- *Storage of classical data:* as mentioned previously, works such as [29,30] have used a PQC-based QRAM circuit to store classical data such as data from a normal distribution, images, and binary data into quantum Hilbert space by training the PQC in a similar way to a machine learning model.

## 4. Practicality of QRAM

Follow-up papers [25,26] have provided possible physical implementations of bucket-brigade QRAM and fan-out QRAM. We first explain these implementations, followed by implementation details on FF-QRAM, qudit-based storage, and trainable PQC-based QRAM. We present a detailed tabular comparison of different QRAM approaches in Table 4.

### 4.1. Bucket-Brigade QRAM Implementation

**Figure 10:**
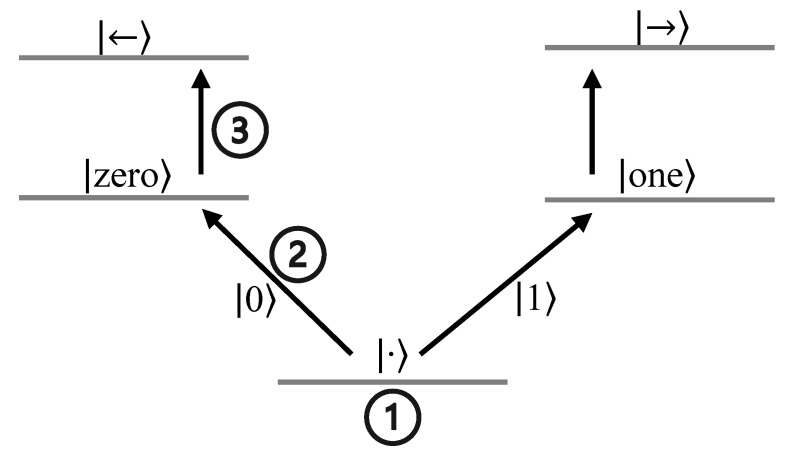
Energy levels of trapped atom-based qutrit switches in bucket-brigade QRAM. **①** Initialized state of qutrit. **②** First incoming photon being absorbed, changing the state of the qutrit, and routing it in either the |zero〉 or |one〉 direction based on the state of the qubit encoded in the photon. **③** Subsequent photons being absorbed into |←〉 or |→〉 and remitted to the next qutrit based on the state of the previous qubit.

To physically implement bucket-brigade QRAM, the authors of [26] incorporated (i) address qubits in the input register as photons that can be sent sequentially, and (ii) qutrits as trapped atoms inside cavities. The qubits encoded in the photons traverse the cavity by encountering the trapped atom-based qutrits. The three states of the qutrits are realized as three different energy levels, with the |·〉 being the lowest energy state along with two higher energy levels: |zero〉, coupled along the left spatial path, i.e., with the left qutrit along the bifurcation graph, and |one〉, coupled along the right spatial path with the right qutrit. This coupling is represented using further higher energy states |←〉 (for left spatial path) and |→〉 (for right spatial path). The energy diagram of these qutrit switches is shown in Figure 10.

Initially, all the qutrits are initialized to the lowest energy state |·〉. When the first photon traverses the cavity and reaches the root node switch, it is absorbed into the higher energy state of the qutrit, either |zero〉 or |one〉, thereby changing the state of the qutrit depending on the quantum state encoded in the photon. This process is often referred to as the Raman transition, where a photon is scattered by a molecule, resulting in a change in the energy of the photon and the vibrational state of the molecule [52]. This is achieved with the help of strong laser fields [53] that help in changing the state of the qutrit from |·〉 to |zero〉 if the photon state is |0〉 and from |·〉 to |one〉 if the photon state is |1〉. After this, when the second photon arrives at the root node switch it is again absorbed and undergoes a Raman transition, this time either from |zero〉 to |←〉 or from |one〉 to |→〉, and is remitted to the qutrit along the correct spatial path based on the state of the qutrit (|zero〉, left path; |one〉, right path). In this way, all the photons of the input register set the qutrit switches one by one until a path from the root node switch to the desired memory cell is created. The output register then either loads contents from the memory cell or stores new data in it via the created path of qutrit switches. When the load/store operation is complete, all the qutrits sequentially undergo a final Raman transition, starting from the last node to the root node, to return to the |·〉 state.

A recent work [47] proposed a quantum circuit-based implementation of bucket-brigade QRAM. For *n* address lines, the quantum circuit requires O(*n*) qubits for the address, O($2^{n}$) ancillary qubits to incorporate the quantum switches, O($2^{n}$) qubits for memory cells, and one qubit for the readout of the memory cell. Figure 4 shows an implementation of quantum circuit-based bucket-brigade QRAM for two address lines and four memory cells in which the memory cell in address |01〉 is being accessed. First, $|a_{1}\rangle =|0\rangle$ changes the state of the first ancillary qubit, which then changes the state of the next ancillary qubit. Based on the output, the path is then routed to the left child switch, where $|a_{0}\rangle =|1\rangle$ is used to switch the right ancillary qubit to the |1〉 state. Finally, a set of Toffoli gates with the ancillary qubit as one control and the memory cell qubit as another control are used to perform the readout. Depending on the address state, only the Toffoli gate controlled by the corresponding memory cell is triggered. In this case, the Toffoli gate’s corresponding memory cell $|m_{01}\rangle$ is triggered to perform a readout operation on the readout qubit.

### 4.2. Fan-Out QRAM Implementation

Two implementations, namely, optical implementation and phase gate implementation, have been proposed for the fan-out QRAM [26]. Understanding the implementations in the original paper may be challenging, as it assumes knowledge of optical and cavity-based quantum systems.

In the phase gate implementation, (i) the address qubits in the input register are photons and (ii) the quantum switches are photonic qubits trapped inside microwave cavities. Overall, for *n* address qubits there are O($2^{n}$) microwave cavities, with each cavity containing a photonic qubit. As mentioned earlier, the $k^{th}$ index qubit fans out and controls $2^{k}$ quantum switches. This is achieved using conditional phase shifters. The MSB address qubit only polarizes the root node photon inside the microwave cavity via the conditional phase shifter attached to it. The next address qubit polarizes two child photons through a conditional phase shifter. This continues until all of the photons inside the microwave cavity are polarized. As a result of this, a resonant path is created from the root cavity to the desired memory cell. Each memory cell consists of two superconducting qubits such that one is for storing information and one is for extracting information. Using a SWAP gate, the contents of the memory cell are then transferred back to the output register through an outgoing photon from the memory cell with the help of the extraction qubit.

Next, we discuss the optical implementation. Here, (i) the address qubits are atoms trapped in a magneto-optical trap and (ii) the quantum switches are photonic qubits that hit the trapped atomic address qubits one-by-one. When the first quantum switch photon hits the first address qubit inside the trapped atom, the trapped atom acts as a controller for changing the polarization state of the photon. This photon then passes through a polarization beam splitter and a half-wave plate to transfer this information to another spatial degree of freedom and create two spatial modes. The two spatial modes are two photonic quantum switches for the next address qubit. Again, each spatial mode transfers the state of the address qubit through a change of polarization and creates two new modes. This continues until $2^{n}$ spatial modes are created, one for each memory cell. Out of these, only one spatial mode is active depending on the address, and the contents of the desired memory cell corresponding to the active spatial mode are swapped out with the contents of the output register using a SWAP gate.

### 4.3. EQGAN QRAM Implementation

Because EQGAN QRAM is a quantum circuit-based QRAM, it can be implemented on superconducting and trapped ion qubits. The authors of [30] implemented EQGAN QRAM on 5 qubits of Google’s Sycamore superconducting processor such that the readout qubit (the top qubit in Figure 7) was the center physical qubit and the rest of the qubits were physically coupled with the readout qubit in the shape of a (+) sign on a grid of qubits.

### 4.4. Qudit Implementation

Qudits are implementable on physical quantum systems that have an infinite spectrum of states, such as superconducting qubits (magnetic flux spectrum) [54], trapped ion qubits (energy band spectrum) [55], and Orbital Angular Momentum (OAM spectrum)-based photonic qubits [56]. For example, in bucket-brigade QRAM the qutrit switches are implemented using trapped atoms in a cavity, with the |·〉 state at a lower energy level and the |zero〉 and |one〉 states at a higher energy level.

### 4.5. Approximate PQC-Based QRAM and Flip-Flop QRAM Implementations

Similar to the EQGAN QRAM, because both of these QRAM approaches are quantum circuit-based they can be implemented on superconducting and trapped ion qubits. As the quantum circuit is known, the QRAM architectures can be replicated on known quantum computing platforms such as Qiskit [57] from IBM, Pennylane [58] from Xanadu, IonQ [59], and many more. Users can replicate the quantum circuit and send either it for simulation on a noiseless/noisy simulator (better for approximate PQC-based QRAM [29], as it is iterative) or run it on actual quantum hardware (better for FF-QRAM [27], as it is non-iterative).

## 5. Challenges and Future Direction

In this section, we examine the current limitations and future directions of various QRAM architectures along with their related challenges. Among the common challenges are:

- *Scalability:* a major challenge in QRAM designs due to constraints in terms of qubit interactions, quantum memory, and coherence. Increasing memory elements in the bucket-brigade, fan-out, and FF-QRAM approaches lead to exponential growth in circuit width and depth. Thus, scalability remains a significant hurdle for large-scale QRAM implementations.
- *Noise Resilience:* a crucial challenge in QRAM architectures, as quantum systems are sensitive to environmental noise. In various QRAM types, increasing memory elements results in a higher circuit depth and qubit count, making the system more vulnerable to noise. Bucket-brigade QRAM is comparatively more resilient to noise than fan-out QRAM [45], while FF-QRAM is susceptible to noise as the number of address lines increases. While PQC-based QRAM has a constant circuit depth, it remains prone to noise-related errors that affect performance on real hardware compared to simulations.
- *No-Cloning Theorem:* the no-cloning theorem [42,60] is a fundamental quantum mechanics principle that prohibits exact copying of unknown quantum states, which poses challenges for various QRAM designs. In bucket-brigade and fan-out QRAM, the theorem limits duplication of quantum states during memory readout. Although solutions such as CNOT and SWAP gates are available, the no-cloning theorem complicates error correction and redundancy schemes in most QRAM designs, presenting a significant challenge [23].
- *Instability of Qudits:* qudit instability primarily affects qudit-based quantum memory, where the qudits are quantum systems with d>2 levels. While qudit-based memory can store more information than qubit-based systems, higher qudit states are unstable and prone to errors [61,62]. For example, the energy gap between higher states in superconducting qubits is less [1]. While this issue is not directly relevant to qubit-based QRAM designs, incorporating qudits for increased storage would introduce similar challenges related to qudit instability.
- *Limited Applicability:* certain QRAM architectures face limitations due to their novelty, experimental difficulties, or specific focus. For example, FF-QRAM has limited applicability beyond quantum forking due to its targeted design. Similarly, qudits face challenges arising from limited research and their increased complexity compared to qubits. Addressing these challenges is essential in order to advance broader applications in quantum computing.

While current QRAM designs face these challenges, ongoing research strives to overcome them. Several recent works have made significant progress in the implementation of bucket-brigade QRAM. In one recent study [63], researchers constructed a circuit implementation of the aforementioned QRAM, demonstrating that when used with classical data it can quickly and repeatedly prepare arbitrary quantum states when the data are already present in memory. Another work [64] discussed the parallelization of queries in bucket-brigade QRAM, showing that the parallelization method is compatible with surface code quantum error correction. In theory, fault-tolerant bucket-brigade QRAM queries can be performed at speeds comparable to classical RAM. A separate article [47] addressed the robustness of bucket-brigade QRAM, revealing that when quantum error correction is applied to the bucket-brigade QRAM circuit, the circuit loses the advantage of having a small number of active gates, as the error correction operates on all of its components.

The precise evaluation of the hardware expenditure associated with QRAM designs, especially in the realm of fault-tolerant systems, may constitute a significant subject of forthcoming research [23]. It is reasonable to anticipate that, compared to conventional surface code implementations [65], the hardware expenses and intricacy will be substantially reduced owing to the noise resilience inherent in bucket-brigade QRAM and the implementation of low-overhead fault tolerance techniques utilizing qubits. While there have been notable advancements in hardware efficiency, in the near future it remains a challenge to develop a QRAM capable of addressing millions or billions of individual memory elements. Exploring applications in which smaller QRAMs can provide value and conducting tailored resource estimations for these use cases could be a key to future progress and development.

## 6. Conclusions

Quantum Random Access Memory (QRAM) serves as a specialized form of memory that enables direct access and manipulation of quantum states, thereby facilitating expedited and efficient data retrieval and storage within quantum systems. Unlike conventional RAM structures that store information in classical bits, (which are incompatible with quantum systems), QRAM operates on the principles of quantum computing. This enables QRAM to store and manipulate quantum data effectively, resulting in considerable acceleration of known quantum algorithms. This review provides a thorough assessment of QRAM, emphasizing its importance and practicality within the context of contemporary quantum computing. We explain the fundamentals of quantum computing and conventional RAM before delving into the foundations of QRAM. Five notable types of QRAM designs are outlined: bucket-brigade QRAM, fan-out QRAM, flip-flop QRAM, qudit-based quantum memory, and approximate PQC-based QRAM. By comparing these diverse architectural approaches and carefully analyzing their implementation, the feasibility of QRAM is thoroughly explored. This analysis concludes by discussing the primary challenges and future directions associated with QRAM development.

## Figures and Tables

**Figure 5:**
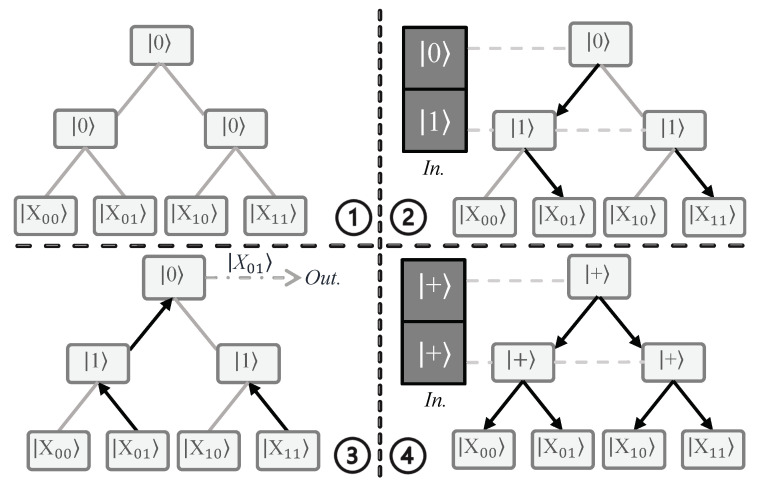
Workings of fan-out QRAM. **①** Fan-out QRAM initialization with all quantum switches initialized to the |0〉 state. **②** Address qubits in the input register control the state of their respective quantum switches and create a path from the root node switch to the desired memory cell $X_{01}$. **③** The data in memory cell $X_{01}$ are accessed in the output register through the active route of the switches. **④** The superposition of addresses means that all routes are switches on, allowing access to the contents of all memory cells.

**Table 1:** A comparison of classical RAM and quantum RAM.

Attributes	Classical RAM	Quantum RAM
*Information storage*	Classical bits (0/1)	Qubits (|ψ〉=α|0〉+β|1〉)
*Access mechanism* *implementation*	Using transistors and capacitors	Encoding into superposition
*Read operation*	Read signal	Quantum swap operation
*Write operation*	Write signal	Qubits in input register
*Gate activations*	Θ($2^{n}$); n=#bits	Θ(*n*); n=#qubits
*Error correction*	Repetition codes	Surface codes
*Scalability*	Increasing #bits	Increasing #qubits

**Table 2:** Creation of dataset with rotation angle for FF-QRAM.

Address (A)	Data (X)	Data Value	Normalized Value ($X_{N}$)	θ= 2arcsin($X_{N}$)
00	$x_{00}$	2(10)	$2/\sqrt{15}=0.51$	1.06
01	$x_{01}$	3(11)	$3/\sqrt{15}=0.77$	1.74
10	$x_{10}$	1(01)	$1/\sqrt{15}=0.25$	0.5
11	$x_{11}$	1(01)	$1/\sqrt{15}=0.25$	0.5

**Table 3:** Truth table for 2-3-1 compression scheme [28].

A	B	C	A’	B’	C’
0	0	0	0	0	0
0	0	1	2	2	0
0	1	0	0	1	0
0	1	1	0	2	0
1	0	0	1	0	0
1	0	1	2	1	0
1	1	0	1	1	0
1	1	1	1	2	0

**Table 4:** Table showing comparison between different QRAM technologies.

*Feature/QRAM*	Bucket-Brigade QRAM [25]	Fanout QRAM [26]	Flip-Flop QRAM [27]	Qudits-Based Memory [28]	Approximate PQC-Based [29] & EQGAN QRAM [30]
*Structure*	Bifurcation graph	Bifurcation graph	Quantum circuit	Higher states	Parametric Quantum Circuit
*Circuit width* (n=#*address* *lines*)	O($2^{n}$)	O($2^{n}$)	O(*n*)	Dependent on *d* (# qudit states)	O(*n*)
*Circuit depth* (n=#*address* *lines*)	O($2^{n}$)	O($2^{n}$)	O($2^{n}$)	Dependent on *d* (# qudit state)	O(1)
* Unique qualities*	Qubits are routed in a sequential fashion	Qubits controlling exponential quantum switches	Quantum circuit-based	Reduces requirements of ancillary qubits to 0	Can be trained similarly to a machine learning model
*Implementation* *technology*	Photons, trapped atoms	Photons, microwave cavities	Superconducting qubits, trapped ion qubits	Superconducting qudits, trapped ion qudits, OAM photonic qudits	Superconducting qubits, trapped ion qubits
* Drawbacks*	Exponential circuit width and depth	Exponential circuit width and depth, susceptible to decoherence	Exponential circuit depth	Unstable higher states	Performance degradation under noise (approx. QRAM), store only simple dataset (EQGAN)

## Data Availability

No new data were created or analyzed in this study. Data sharing is not applicable to this article.
